# Collared versus collarless hydroxyapatite-coated stems for primary cementless total hip arthroplasty; a systematic review of comparative studies. Is there any difference in survival, functional, and radiographic outcomes?

**DOI:** 10.1051/sicotj/2024003

**Published:** 2024-02-15

**Authors:** Vasileios Giovanoulis, Eustathios Kenanidis, Florence Aïm, Zakareya Gamie, Simon Marmor, Michael Potoupnis, Sébastien Lustig, Eleftherios Tsiridis

**Affiliations:** 1 Orthopedic Surgery Department, Groupe Hospitalier Diaconnesses Croix Saint-Simon 125 Rue d’Avron 75020 Paris France; 2 Academic Orthopaedic Department, Aristotle University Medical School, General Hospital Papageorgiou Ring Road Efkarpia Thessaloniki 56403 Greece; 3 Center of Orthopaedic and Regenerative Medicine (CORE), Center for Interdisciplinary Research and Innovation(CIRI)-Aristotle University of Thessaloniki (AUTH), Balkan Center Buildings A & B, Thessaloniki, 10th km Thessaloniki-Thermi Rd PO Box 8318 GR 57001 Greece; 4 Orthopaedics Surgery and Sports Medicine Department, FIFA Medical Center of Excellence, Croix-Rousse Hospital, Lyon University Hospital, Hospices Civils de Lyon 103 Grande Rue de La Croix Rousse 69004 Lyon France

**Keywords:** Total Hip Arthroplasty (THA), Corail, Stem, Collar, Outcomes

## Abstract

*Introduction*: This systematic review aims to critically assess the literature comparative studies investigating collared and collarless Corail stem in primary total hip arthroplasty (THA) to find differences in revision rates, radiographic and clinical outcomes, and postoperative complications between these two types of the same stem. *Methods*: Eligible studies were found by searching PubMed, Science Direct/Scopus, and the Cochrane Database of Systematic Reviews from conception till May 2023. The PRISMA guidelines were followed. The investigation encompassed randomized controlled trials, case series, comparative, cohort, and observational studies that assessed at least one comparative outcome or complication between collared and collarless Corail stems. *Results*: Twelve comparative studies with 90,626 patients undergoing primary THA were included. There were 40,441 collared and 58,543 collarless stems. The follow-up ranged from 12 to 360 months. Our study demonstrated no significant difference in stem revision relative risk (RR = 0.68; 95% confidence interval (CI), 0.23, 2.02; *p* = 0.49), number of radiolucent lines (RR = 0.3; 95% CI, 0.06, 2.28; *p* = 0.29) and overall complication risk (RR = 0.62; 95% CI, 0.22, 1.76; *p* = 0.37) between collared and collarless stems. The collared stems demonstrated significantly lesser subsidence (mean difference: 1.01 mm; 95% CI, −1.77, −0.25; *p* = 0.009) and risk of periprosthetic fractures (RR = 0.52; 95% CI, 0.29, 0.92; *p* = 0.03). *Conclusion*: The comparative studies between collared and collarless stem groups showed similar survival and overall complication rates and functional outcomes. The similar revision rates between groups make the impact of higher subsidence for collarless stems uncertain. The lower risk of periprosthetic fractures in the collared stems group must be clarified further but could be related to increased rotational stability.

## Introduction

Total hip arthroplasty (THA) stands as a successful procedure for hip osteoarthritis and over time, technological advancements, including different types of cementless fixation and design philosophies, have expanded the implant options, indications, and age-group target [1–5]. The Corail stem^®^ (DePuy Synthes) is a non-cemented, fully hydroxyapatite-coated femoral stem made of forged titanium alloy for hip reconstruction. It was introduced in the late 1980s and has excellent long-term published outcomes [6]. The basic stem design, combining surface finish and full hydroxyapatite coating, prevents the release of metal ions and promotes osseointegration with the endosteal surfaces [7]. The Corail stem has collarless and collared options. The collarless one has standard and high offset stem options for proper soft tissue tensioning and femoral offset restoration. The collared stem is available in standard and coxa-vara offset versions and is mainly used for patients with poor bone quality.

The Norwegian Joint Registry recently supported that the collared Corail stem has shown reduced revision risk, significantly adjusted health gain, and fewer revisions due to pain, periprosthetic fractures (PPFs), and dislocations compared to the standard collarless stem [8]. Proponents of a collared Corail stem [9, 10] support that this stem benefits the initial implant stability, allows faster postoperative full-weight bearing, protects against stem migration, and beneficially distributes the vertical forces through the collar into the medial calcar [9]. Several studies supported that collared stems might have better survival rates, less risk of stem migration, and comparable functional outcomes than collarless stems [11–13].

This systematic review aimed to ascertain any outcome discrepancies, including survival, functional and radiographic outcomes between collared and collarless hydroxyapatite coated (Corail^®^) stems for primary cementless THA, evaluating only comparative studies between these two types of the same femoral stem.

## Materials and methods

### Search strategy

The present systematic literature review followed the PRISMA (Preferred Reporting Items for Systematic Reviews and Meta-analyses) guidelines [14] and was in line with the protocol agreed by all authors. This comprehensive review protocol was registered in the International Prospective Register of Systematic Reviews (PROSPERO) under CRD42023435176.

The studies were found through extensive search in PubMed, Science Direct/Scopus, and Cochrane Database of Systematic Reviews from conception up to May 2023 for all databases. The following search terms [All Fields] and their MeSH terms alone or in combination using Boolean operators were used: “hip arthroplasty”, “femoral”, “stem”, “cementless”, “Corail” “Hydroxyapatite coated stem”, “collar”, “collared”, “collarless”. The search algorithm that was used: (((((femoral) OR (hip)) OR (stem)) AND ((arthroplasty) OR ("hip arthroplasty"))) AND (((((cementless) OR (uncemented)) OR (coated)) OR ("hydroxyapatite coated")) OR (corail))) AND (((collar) OR (collared)) OR (collarless)). The individual reference lists of the found papers were further screened to ascertain additional cases.

### Eligibility criteria

Randomized controlled trials (RCTs), case series, comparative, cohort, and observational clinical studies that assessed at least one comparative survival, radiological, or clinical outcome between collarless and collared Corail stem groups of adult patients undergoing primary THA were included in this meta-analysis. The cup outcomes were not evaluated. Papers published in English and French with a minimum one-year follow-up were considered eligible for inclusion in the analysis.

Studies reporting outcomes on cemented Corail stem or other than Corail uncemented stem (DePuy Synthes, Warsaw, Indiana^®^) were excluded from the study. Additionally, case reports, narrative or systematic reviews, meta-analyses, letters to the editor, conference proceedings, and in vitro and cadaver studies were excluded.

### Study selection

Two reviewers (V.G., E.K.) searched the literature independently. Initially, the articles were analyzed and selected by title and abstract based on the inclusion criteria. After excluding studies that did not meet the inclusion criteria, full texts of the selected papers were evaluated. Any disagreements between the two authors were resolved via discussion and consensus with a third author (E.T.). Excluded studies and reasons for exclusion are listed in Appendix 1.

### Data extraction and analysis

The same authors (V.G., E.K.) reviewed the papers separately and extracted the data for each included study. They used a predefined Microsoft Excel spreadsheet for data extraction. The following data were extracted: (1) study type details: authors, publication year, country, study design, level of evidence; (2) study population: sample size, age, gender, body mass index (BMI); (3) follow-up; (4) stem offset, surgical approach, preoperative diagnosis; (5) acetabular implant (6) femoral stem survival (endpoint stem revision); (7) radiographic signs: subsidence, translation, stem alignment, migration, radiolucent lines (RLLs); (8) clinical outcomes: preoperative and postoperative Harris Hip Score (HSS), Western Ontario and McMaster Universities arthritis index (WOMAC), Oxford Hip Score (OHS); (9) postoperative complications: mechanical failures, aseptic loosening (AL), infections, dislocations, PPFs, revisions for any reason. When data were missing, attempts were made to email the authors with up to three tentatives. Any discrepancies were resolved through consensus with the senior author.

### Methodological quality assessment

Quality assessment of the included studies was performed using The Cochrane Risk of Bias 2 tool (ROB 2) for RCTs [15] and the Newcastle-Ottawa Scale (NOS) [16] for cohort studies. The NOS evaluates study cohort selection, comparability, and exposure-outcome relationships using a “star rating” system of up to nine stars. The ROB 2 tool assesses the risk of bias in RCTs across five domains where bias may be introduced. Two authors conducted the quality assessment individually, resolving disagreements via consensus.

## Results

### Search results

**Figure 1:**
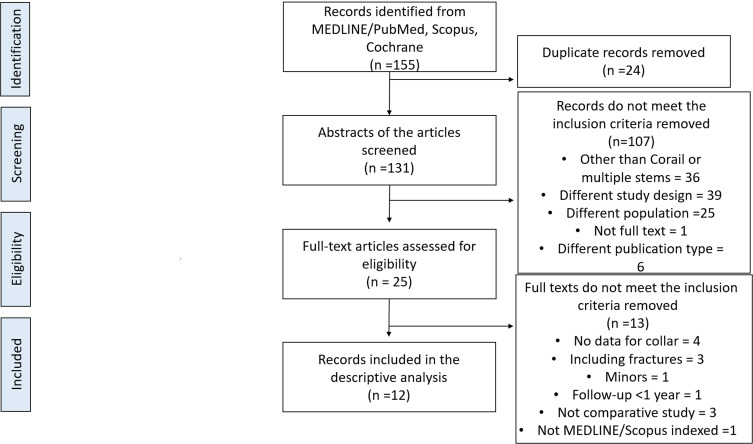
Preferred Reporting Items for Systematic Reviews and Meta-analyses (PRISMA) flowchart.

The initial search identified 155 eligible studies. After removing duplicates, 131 papers were screened based on the titles and abstracts. Twenty-four papers were considered suitable and assessed in full text. When the predefined inclusions and exclusion criteria were applied, 12 studies were finally selected [8, 11, 12, 17–25] (Figure 1).

### Included studies design

The included papers were published between 2016 and 2022. Ten studies [8, 17–25] were retrospectives, and the remaining two [11, 12] were RCTs, respectively. Four studies were conducted in the United Kingdom, two from Canada and France, and one from Australia, Austria, Germany, and Norway. The primary aim of the three studies [12, 21, 23] was survival rates and clinical and radiological outcomes of the Corail Stem. Four papers [17, 18, 20, 25] mainly evaluated stem subsidence and RLLs, while three others [8, 22, 24] focused on stem performance and revision rates. One study [19], mainly assessed PPFs, survival, and complication rates, and another [11], the Corail stem stability.

### Overall patients’ characteristics

A total of 90,626 patients (98,984 stems) undergoing cementless THA with Corail stem were included. There were 40,441 collared and 58,543 collarless stems. The female/male ratio of the included studies was 1.62. The mean patients’ age at the time of the surgery was 68.5. Only one study did not report the overall mean age [22]. BMI was reported in 6 out of 12 studies [11, 12, 17–19, 23]; the mean BMI of the included studies was 27.6 (kg/m^2^) (*SD* = 1.03). The follow-up ranged from 12 to 360 months. Six studies reported extensive follow-ups up to 6 years [8, 19–21, 23, 24]; 2 had notable >10-year follow-ups [20, 23]. A mid-term (2–9 years) follow-up was reported in five studies [8, 17, 19, 21, 24], and a short-term (1–2 years) follow-up in four studies [11, 12, 18, 25]. Table 1 shows the patients’ demographics and study characteristics.

**Table 1:** Demographics and other study characteristics of studies included in the meta-analysis.

Authors	Year	Country	Study type	Level of evidence	Patients	Stems	Sex (female/male)	Mean Age at THA (years) [range]	BMI (kg/m^2^) [range]	Mean follow-up (months) [range]
Dammerer et al. [17]	2022	Austria	RCS	III	105	109	60/45	67.8 [21.6–90.5]	26.8 [17.4–50.8]	25.1 [8–57]
Polus et al. [11]	2022	Canada	RCT	I	79	79	36/43	65.2 [41–85]	28.4 [17.3–38.9]	12
Belgaïd et al. [19]	2022	France	RCS	IV	128	128	89/39	83 [80–93]	26.2 [16.9–38.2]	96 [60–120]
Wirries et al. [18]	2021	Germany	RCS	III	186	186	115/71	68.4 [34.2–89.5]	27.3 [18.6–44.3]	12
Melbye et al. [8]	2021	Norway	RCS	III	43,318	51,212	26,844*/15,923*	65 [54–76]*	/	92.4 [12–360]
Karayiannis et al. [20]	2021	UK	RCS	III	288	288	167/121	70 [49–83]	/	130 [120–140]
Perelgut et al. [12]	2020	Canada	RCT	I	58	58	20/29	64.6 [56.5–72.7]	29.2 [24.4–33.1]	13
Magill et al. [21]	2019	UK	RCS	III	636	636	351/285	63.5 [58–68]	/	72 [62.4–81.6]
Hoskins et al. [22]	2019	Australia	RCS	III	41,265	41,265	/	/	/	/
Louboutin et al. [23]	2017	France	RCS	IV	133	140	55/85	69 [35–92]	27 [16–39]	120 [36–144]
Magill et al. [24]	2016	UK	RCS	III	4309	4802	2716/2086	70 [62–76]	/	72 [12–127]
Al-Najjim et al. [25]	2016	UK	RCS	III	121	121	66/51	67.1 [38–88]	28 [22–31]	12

THA: Total Hip Arthroplasty, UK: United Kingdom, BMI: Body Mass Index, kg: kilogram, m: meter, RCS: retrospective comparative study, RCT: randomized controlled trial.

* Data from 2008 to 2018.

### Surgical data

Eight studies reported the preoperative diagnosis for primary THA [8, 11, 12, 17–19, 22, 23], and then showed the surgical approach used [8, 11, 12, 17–21, 23, 24]. The preoperative diagnosis was osteoarthritis in 62,336 patients (68.8%). The posterior approach was used in 22,524 (24.9%), the anterolateral in 13,854 (15.3%), and the anterior in 11,033 cases (12.1%); the surgical approach was not reported in 43,029 patients (47.5%). There were 40,441 collared stems (40.8%) and 58,543 collarless (59.2%) examined. There were 13,335 standard offsets with collar, 29,994 standard offsets without collar, 6148 high-offset without collar, 1025 high-offset with collar, 6947 Coxa vara with collar, and there were no available offset design data for 41,559 stems. Eight studies [11, 12, 17, 18, 20, 21, 24, 25] used the Pinnacle (PINNACLE^®^ Hip Solutions, DePuy Synthes^©^), one study [19] the Sunfit (Cotyle Novae^®^), and another [23] the Allofit^®^ and Lagoon^®^ cup. Two authors provided no cup information [8, 22]. Detailed knowledge of surgical data is presented in Table 2.

**Table 2:** Operative and implant characteristics of the included studies in the meta-analysis.

Authors	Stem type		Stem offset						Cup	Approach				Preoperative diagnosis		
	Collared stems	Collarless stems	KHO, high offset without collar	KS, standard offset without collar	Coxa vara with collar	KA, standard offset with collar	KLA, high offset with collar	Other, NR		A	P	ARL	Other, NR	OA	AVN	Dysplasia
Dammerer et al. [17]	85	24	13	11	7	32	40	6	Pinnacle	107	2	0	0	104	4	1
Polus et al. [11]	36	43	0	43	0	36	0	0	Pinnacle	49	0	30	0	79	0	0
Belgaïd et al. [19]	117	11	11	0	0	98	19	0	Sunfit	0	128	0	0	121	1	2
Wirries et al. [18]	146	40	/	/	/	/	/	186	Pinnacle	94	0	92	0	186	0	0
Melbye et al. [8]	17,275	33,937	5009	28,928	6924	10,351	0	0	/	10,725*	16,546 *	13,732*	1,764*	20,391*	/	/
Karayiannis et al. [20]	99	189	100	89	0	99	0	0	Pinnacle	0	288	0	0	/	/	/
Perelgut et al. [12]	19	22	0	22	0	19	0	0	Pinnacle	58	0	0	0	58	0	0
Magill et al. [21]	318	318	159	159	0	161	157	0	Pinnacle	0	636	0	0	/	/	/
Hoskins et al. [22]	18,936	22,310	/	/	/	/	/	41,246	/	/	/	/	41,265	41,265	0	0
Louboutin et al. [23]	28	112	0	112	0	28	0	0	Allofit, Lagoon	0	140	0	0	132	4	4
Magill et al. [24]	3316	1482	856	626	/	2507	809	/	Pinnacle	0	4802	0	0	/	/	/
Al-Najjim et al. [25]	66	55	/	/	/	/	/	121	Pinnacle	/	/	/	/	/	/	/
Total	40,441	58,543	6148	29,990	6931	13,331	1025	41,559	/	11,033	22,542	13,854	1764	62,336	9	7

NR: not reported, A: Anterior, P: Posterior, ALR: Antero-lateral, OA: Osteo-arthritis, AVN: Avascular necrosis, KHO: high offset stem without collar, KS, standard offset stem without collar, KA: standard offset stem with collar, KLA: high offset stem with collar.

* Data from 2008 to 2018.

### Survival rate

**Figure 2:**
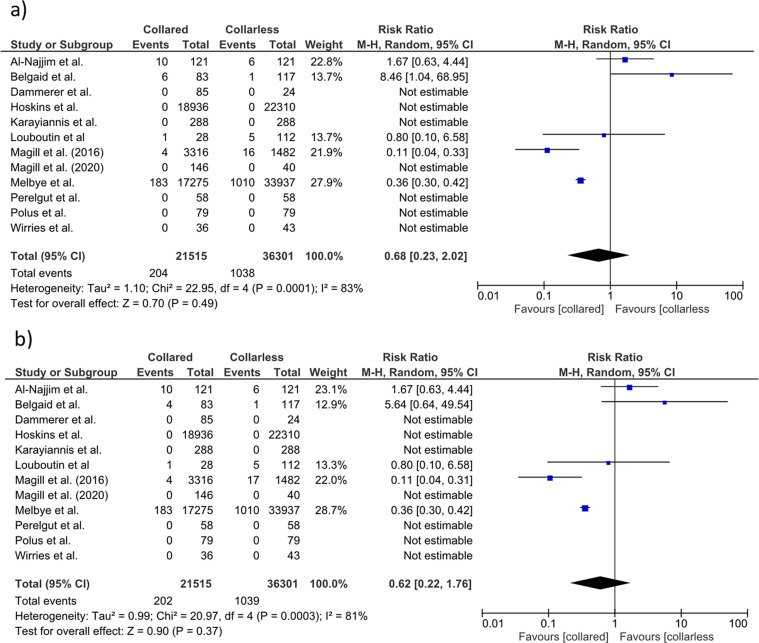
Forest plot for comparison of (a) stems revisions and (b) overall complications between collared and collarless stems.

Eight authors provided data on the stem survivorship rate [8, 11, 12, 17, 18, 20–22]. Seven studies [11, 12, 17, 18, 20–22] reported excellent (100%) survivorship in both types of stems at a mean follow-up of 42.5 months. One study [8] reported that standard collared stems had a significantly better survival rate for any reason compared to the standard collarless (99.0% (95% CI, 98.8, 99.2) vs. 97.6% (95% CI, 97.2, 98.0)). The same study [8] also demonstrated that the collared stems showed significantly lower revision rates for AL (99.1% (95% CI, 98.9, 99.3) vs. 99.7% (95% CI, 99.5, 99.9)) and for PPFs (99.4% (95% CI, 99.0, 99.8) vs. 98.6% (95% CI, 98.4, 98.8)) than the non-collared stems. The rest of the studies did not assess the survival rates or provide available information concerning the stem revision [19, 23–25]. Our meta-analysis demonstrated a similar stem revision relative risk (RR) between collared and collarless stems (RR = 0.68; 95% CI, 0.23, 2.02; *p* = 0.49). The heterogeneity of the included studies was considerable (83%) and statistically significant. Figure 2 depicts the forest plot of stem revisions.

### Radiographic outcomes

**Figure 3:**
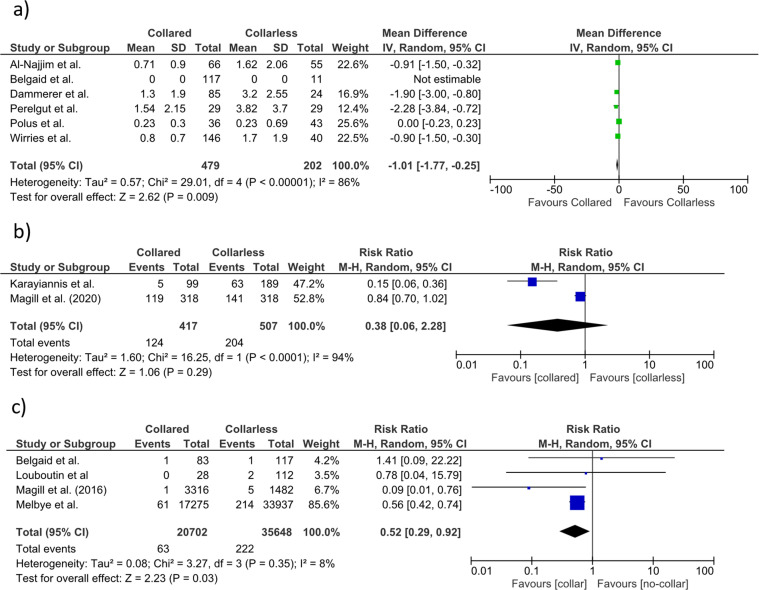
Forest plot for comparison of (a) subsidence, (b) RLLs, and (c) PFFs between collared and collarless stems in the included studies.

Six studies [11, 12, 17–19, 25] reported the distance in millimetres (mm) of stem subsidence, and four [19, 20, 24, 25] evaluated the presence of RLLs. Three studies [11, 19, 25] found no significant difference in subsidence measurements at 12 [25], 13 [11], and 96 months [19]. One author found significantly higher mean subsidence by 1.9 mm of uncollared stems at 18 months [17]; another study [12] showed a significant difference with a mean difference of 2.28 mm at 13 months. Finally, one study [18] found a substantial difference in the mean difference of subsidence by 0.9 mm at 12 months. The overall mean subsidence of the collared stems was 0.87 mm against 2.07 mm of the non-collared stems. The collarless stems demonstrated a significantly greater overall mean subsidence of 1.01 mm than the collared stems (95% CI, −1.77, –0.25; *p* = 0.009). The *I*^2^ index of the studies that assess the subsidence was 86%. Figure 3 illustrates the forest plot of the reported stem subsidence of the included studies.

Our meta-analysis of two studies [20, 21] comparing collared versus collarless stems showed nonsignificantly different RR for the presence of RLLs (RR = 0.38; 95% CI, 0.06, 2.28; *p* = 0.29, *I*^2^ = 94%) (Figure 3). Regarding the stem alignment, two studies [12, 18] found no significant malalignment at 12 [18] and 13 [12] months. Only one study, [11], found that collarless stems presented significantly more valgus/varus rotation at 13 postoperative months.

### Functional outcomes

Two studies assessed the postoperative WOMAC score [12, 18]. One study [12] demonstrated significantly greater WOMAC and short form 12 mental and physical outcomes favoring collared stems, while the University of California/Los Angeles activity score was comparable between groups [12]. One study (8.3%) [11] investigated the timed-up-and-go test and the average daily step count with no significant differences between groups. Three studies evaluated the OHS with no available comparative data.

### Postoperative complications

Six out of twelve studies reported the risk of postoperative complications [8, 19, 22–25]. Four papers focused on PPFs [8, 19, 23, 24]. Our meta-analysis demonstrated that postoperative complication RR was not significantly diminished in patients with collared compared to non-collared Corail stems (RR = 0.62; 95% CI, 0.22, 1.76; *p* = 0.37). The heterogeneity of the included studies was high (81%) and statistically significant (Figure 2). Table 3 presents the details of the overall complications. The included studies’ meta-analysis showed that the incidence of PPFs was significantly lower for the collared than the non-collared stems (RR = 0.52; 95% CI, 0.29, 0.92; *p* = 0.03) with no significant heterogeneity (*I*^2^ = 8%) (Figure 3).

**Table 3:** Postoperative complications of the patients in the included studies.

Authors	Complication	Collared (*N*, %)	Collarless (*N*, %)	Overall (*N*, %)	*p*-value
Dammerer et al. [17]	Any	0	0	0	–
Polus et al. [11]	Any	0	0	0	–
Wirries et al. [18]	Any	0	0	0	–
Belgaïd et al. [19]	Periprosthetic fracture	1 (0.8%)	1 (9%)	2 (1.6%)	n.s.
	Distal femoral fracture	1 (0.8%)	0	1 (0.8%)	
	Infection	2 (1.6%)	0	2 (1.6%)	
Melbye et al. [8]	Revision for any reason	183 (1.06%)	1010 (2.97%)	1193 (2.33%)	<0.001
	Revision for aseptic loosening	61 (0.35%)	163 (0.48%)	224 (0.44%)	
	Revision for PFF	61 (0.35%)	214 (0.63%)	275 (0.54%)	
Karayiannis et al. [20]	Any	0	0	0	–
Perelgut et al. [12]	Any	0	0	0	–
Magill et al. [21]	Any	0	0	0	–
Hoskins et al. [22]		n.a.	n.a.	n.a.	n.a.
Louboutin et al. [23]	PFF	0	2 (1.78%)	2 (1.4%)	n.a.
	Infection	1 (3.57%)	1 (0.9%)	2 (1.4%)	
	Aseptic loosening	0	2 (1.78%)	2 (1.4%)	
	Dislocation	n.a.	n.a.	6 (4.3%)	
	Calcar fracture	n.a.	n.a.	5 (3.6%)	
	Great trochanter fracture	n.a.	n.a.	2 (1.4%)	
	Sciatic nerve palsy	n.a.	n.a.	2 (1.4%)	
	Iliopsoas irritation	n.a.	n.a.	2 (1.4%)	
	Ceramic liner fracture	n.a.	n.a.	1 (0.7%)	
Magill et al. [24]	Instability			22 (0.46%)	n.a.
	Infection			20 (0.42%)	
	Aseptic loosening stem	3 (0.09%)	12 (0.8%)	15 (0.31%)	
	Femoral fracture	1 (0.03%)	5 (0.34%)	6 (0.12%)	
	Metallosis			5 (0.10%)	
	Failure of acetabular component	n.a.	n.a.	3 (0.06%)	
	Liner dissociation			6 (0.12%)	
	Acetabular fracture	n.a.	n.a.	1 (0.02%)	
	Aseptic loosening cup	n.a.	n.a.	1 (0.02%)	
	Leg length discrepancy	n.a.	n.a.	1 (0.02%)	
Al-Najjim et al. [25]	Surgical site infection	4 (6%)	1 (1.8%)	5 (4.13%)	n.a.
	Cellulitis	1 (1.5%)	1 (1.5%)	2 (1.65%)	
	Deep vein thrombosis	2 (3%)	2 (3.6%)	4 (3.31%)	
	Cup revision	1 (1.5%)	1 (1.8%)	2 (1.65%)	
	Stem revision	1 (1.5%)	1 (1.8%)	2 (1.65%)	
	Iatrogenic fracture	1 (1.5%)	0	1 (0.83%)	

*N*: Number, n.a.: not available, n.s.: not significant, PFF: periprosthetic fractures.

### Methodological quality of the included studies

All ten retrospective cohort studies were graded as good/high quality. According to the ROB2 tool, the two RCT studies were rated as “low risk” of bias. Details of the studies’ quality assessment can be accessed in Appendix 2.

## Discussion

The current systematic review is an initial effort to investigate overall differences in outcomes between collared and collarless Corail^®^ stems in patients undergoing primary THA. The work demonstrated that overall revision rate, postoperative complications, and functional outcomes were similar between collared and collarless stems; however, collared stems showed significantly lower mean subsidence and PPFs’ risk. The clinical significance of higher subsidence for collarless stems remains uncertain and further investigation is required to understand the association between having a collar and lowered risk of PPF.

### Survivorship

This meta-analysis did not reveal significantly different stem revision rates between collarless and collared stems, supporting a high survival probability for both stem design types in a long-term follow-up [6, 26, 27]. In this review, over half of the studies showed a 100% survival rate with no complications or revisions for both stem types [11, 12, 17, 18, 20–22]. However, the other included studies in this review did not estimate the revision rates. Only one national register study [8] in our review found the significantly better 10-year performance of standard collared stems over standard collarless in terms of any reason, AL and PPFs. According to the same study [8], 88.4% of the standard collarless stems were free of stem revision at a 30-year follow-up. The largest national registry study reviewed in our analysis [22] conducted a sub-analysis comparing the survival rate between collared and collarless stems and found no significant differences. Despite the higher risk of subsidence for collarless stems, our study found that both stems have similar overall survival rates.

### Radiographic outcomes

The necessity of a collar in uncemented arthroplasty to prevent clinically meaningful subsidence remains a subject of ongoing debate. The meta-analysis of the included studies demonstrated a 1 mm significantly higher subsidence of the collarless stems. The reported subsidence was inconsistent over time, and different radiostereometric or radiographic analyses were used in the included studies. Dammerer et al. [17] used the EBRA-FCA system to demonstrate non-significant subsidence for collarless implants at 12 months post-op but a statistically significant difference between collared and collarless implants only at 18 months. Perelgut et al*.* [12] reported significantly higher subsidence for collarless than collared stems at 13 months follow-up compared to the first postoperative day; however, this difference was insignificant when subsidence was compared to the fifteenth postoperative day. Other reports have previously suggested no significant subsidence difference between collarless and collared stems [18, 25, 28]. The current study indicated that the collar mitigates against subsidence but does not prevent it completely. Collared stem subsidence may be due to difficulty achieving optimal contact between the collar and the calcar. Also, orthopaedic surgeons who use collared stems to prevent subsidence may unconsciously opt for smaller stem sizes in osteoporotic femurs to avoid intraoperative fracture. However, an undersized stem may increase the risk of subsidence and revision due to AL in the long-term [29, 30]. Surgeons using collarless stems may opt for the largest stem size to avoid subsidence, increasing the PPF risk [13, 22]. It remains uncertain whether the higher subsidence for collarless stems has clinical significance, as the overall revision rate of collared and collarless stems is comparable.

### Radiolucent lines (RLLs) and aseptic loosening (AL)

Our meta-analysis found no significant difference in the incidence of RLLs between collared and collarless stems despite less than half of the studies reporting on them. Previous studies have reported RLLs in patients with the Corail stem over a long-term follow-up [31]. Reports suggested that using collared stems could result in better outcomes [21, 24]. A ten-year follow-up study [20] found a significantly higher number of RLL in collared than non-collared stems, regardless of the bearing type. A non-comparative study of 636 Corail stems with a median 6-year follow-up found a significantly lower RLLs prevalence in zone 7 in the collared (2.6%) compared to the collarless stems (23.6%) [21]. This study suggested that using the proper size Corail stem improves stability and promotes osseointegration, preventing the RLLs development in zone 7.

Cadaveric and *in vitro* studies supported that collared stems improve stability and promote osseointegration, especially in osteoporotic patients, by increasing resistance against various forces at the bone/implant interface [9, 32, 33]. However, clinical data are limited. Most implant migration occurs from the day of surgery to 2 weeks after the operation; it stabilizes thereafter, suggesting adequate fixation and a low AL risk in both collared and collarless stems [11]. During a 2-year follow-up, collarless stems showed an initial migration of 0.73 mm in the first 6 months, but no further measurable subsidence was reported in subsequent measurements using radio stereometric studies [34]. Clinically significant subsidence may occur if the stem size is underestimated and full weight bearing is allowed in the initial postoperative period [35]. Both collared and non-collared stems must be implanted at the appropriate size to ensure proper osseointegration. An undersized collared stem cannot rely on a collar’s protective role to osseointegrate [30].

The impact of stress shielding around the calcar region on RLLs remains unclear. It is supported that a stem collar may cause stress shielding in the lesser trochanter cortical bone, increasing calcar resorption and stress levels over time [36, 37]. Finite element analysis showed that conical collars increase stress transfer and reduce micromotion compared to flat collars [38, 39]. The collar’s optimal contact with the calcar is crucial to load the medial cortex properly, minimising bone loss from stress shielding [36]; however, this is a challenging task during surgery [36, 40]. Besides, a collar could generate an impingement within the calcar region during stem subsidence, triggering a cantilever-like motion that could ultimately lead to stem failure [9, 41]. The review did not find evidence of stress shielding, and more radio-stereometric studies may be beneficial.

### Functional outcomes

The authors could not conduct a meta-analysis due to the various functional scores used in the included studies. However, data from recent studies indicate no significant functional differences. An RCT reported no significant physical activity or function differences between collared and collarless stem patients [12]. Karayiannis et al. [20] found no clinical impact on the OHS at a 10-year follow-up despite the significantly higher RLLs presence in collarless than collared stems. Magill et al. reported favorable outcomes in unrevised Corail stems, regardless of the presence of RLLs or collar [21]. Another study demonstrated favorable HHS and visual analogue scales for both stem types, even in patients over 75 years old [42].

### Overall complications

This meta-analysis did not find significant differences in overall complication rates between stem types; however, collared stems showed a significantly lower PPF incidence. Limited reports have supported that a potential protective collar function can significantly reduce the AL incidence in a long-term follow-up [8, 43]. However, AL is a multifactorial process that may be influenced by various factors throughout the stem lifespan [24, 43–45]; therefore, it cannot be thoroughly evaluated in the long-term follow-up. Our study found no AL risk difference between collared and collarless Corail stems, indicating the need for further studies.

The current study demonstrated that collared Corail stems had a lesser PPF risk than collarless ones. The recent Norwegian registry study [8] reported significantly lower PPF rates for the collared than collarless Corail stems in a long-term follow-up. It is important to exercise caution when interpreting these findings. Biomechanical, cadaveric studies and meta-analyses have shown that collared stems provide increased implant stability due to reduced rotational and varus forces on the bone-implant interface [9, 27, 46]. As a result, collared stems are primarily utilized in elderly patients with osteoporotic femurs, as they are thought to offer protection against subsidence and early failures [47, 48]. However, it is difficult to explain the reduced PPF risk. Most early postoperative PPFs occur intraoperatively, while late PPFs are mainly osteoporotic, depending on bone quality. When using an uncemented collarless stem, surgeons may opt for the largest possible stem size to achieve the best primary stability. However, this may increase the PPF risk, which could partly explain the difference in PPF rates between collared and non-collared stems. On the other hand, a surgeon using a collared, uncemented stem could choose a slightly smaller stem size and still rely on the collar to prevent subsidence. Additionally, this approach could potentially lower the PPF risk [30]. Besides, the collared and collarless stems were traditionally used for different age populations, making direct comparisons between these two stem groups in registry results challenging. Collared stems were mainly used for the elderly population or patients with distinct anatomical characteristics according to Dorr’s classification [49, 50]. Further clarification regarding the lower risk of PPFs in the collared stem group is needed.

### Limitations

Our study has limitations. Firstly, the included papers were of low quality, primarily consisting of retrospective studies. Secondly, heterogeneity among studies and measurement bias may affect the meta-analysis interpretation due to variations in definitions and outcome reporting. Additionally, the analysis did not consider bearing surfaces, age/sex grading, or differences in follow-up periods, countries, and surgical approaches that could affect reoperation rates. However, the strength of the present work is that we analyzed a substantial number of stems following the appropriate methodology provided by the PRISMA guidelines.

## Conclusions

In a long-term follow-up, both collared and collarless Corail stems showed excellent survivorship rates, similar radiographic and functional outcomes, and overall complication rates. Collared stems offer better protection against subsidence, but the clinical significance of this is still unclear and requires further evaluation. The lower risk of PPFs in collared stem cases may be due to increased rotational stability but should be further clarified. The decision to use a collared stem remains dependent on the surgeon’s preferences, and the need for more studies in the future is evident.

## Funding

This research did not receive any specific funding.

## Conflict of interest

The authors declare that they have no relevant financial or non-financial interests related to this work.

VG, EK, FA, ZG, SM, MP: Declare that they have no conflict of interest.

SL: Consultant for Stryker, Smith and Nephew, Heraeus, DePuy Synthes. Institutional research support to Lepine and Amplitude. Editorial Board for the Journal of Bone and Joint Surgery (Am).

ET: Editorial Board for the Journal of Arthroplasty.

## Informed consent

This article does not contain any studies involving human subjects.

## Authors contribution

V. Giovanoulis: Conceptualization, methodology, data collection, writing – original draft.

E. Kenanidis: Data collection, writing, reviewing, and editing.

F. Aim: Conceptualization, data collection, writing, reviewing.

Z. Gamie: Conceptualization, writing, reviewing, and editing.

S. Marmor: Writing, reviewing, and editing.

M. Potoupnis: Conceptualization, supervision, validation, writing, reviewing, and editing.

S. Lustig: Supervision, writing, reviewing, and editing.

E. Tsiridis: Conceptualization, methodology, data curation, supervision, validation, writing, reviewing, and editing.

## Supplementary material

The supplemental material is available at https://www.sicot-j.org/10.1051/sicotj/2024003/olm. *Appendix 1*: List of studies excluded and reasons for exclusion. *Appendix 2*: Quality of included studies assessed by New-castle Ottawa scale.
