# Supplementary material for: Collared versus collarless hydroxyapatite-coated stems for primary cementless total hip arthroplasty; a systematic review of comparative studies. Is there any difference in survival, functional, and radiographic outcomes?
Source: SICOT J. 2024 Feb 15;10:8. doi: 10.1051/sicotj/2024003 (PMC10868518; doi:10.1051/sicotj/2024003)
Supplement: Supplementary file 1 — Appendix 1: List of studies excluded and reasons for exclusion. [file sicotj-10-8-s1.pdf]

Appendix 1

Title	Authors	Raisons
Risk of subsidence and peri-prosthetic fractures using collared hydroxyapatite-coated stem for hip arthroplasty in the elderly	Syed F.; Hussein A.; Katam K.; Saunders P.; Young S.K.; Faisal M. et al.	Not comparative study
Cementless lateralized stems in primary THA: Mid-term survival and risk factors for failure in 172 stems	Courtin C.; Viste A.; Subtil F.; Cantin O.; Desmarchelier R.; Fessy M.H. et al.	Not comparative study
Fully hydroxyapatite-coated collared femoral stems in direct anterior versus direct lateral hip arthroplasty	Syed F.; Hussein A.; Katam K.; Saunders P.; Young S.K.; Faisal M. et al.	Not comparative study
Mid-term progressive loosening of hydroxyapatite-coated femoral stems paired with a metal-on-metal bearing	Gascoyne T.; Flynn B.; Turgeon T.; Burnell C. et al.	No data for collar
Uncemented total hip arthroplasty can be used safely in the elderly population	Lewis P.M.; Khan F.J.; Feathers J.R.; Lewis M.H.; Morris K.H.; Waddell J.P. et al.	No data for collar
The intraoperative use of a calliper predicts leg length and offset after total hip arthroplasty. Component subsidence influences the leg length	Fansur M.; Yurdi N.A.; Stoewe R. et al.	No data for collar

Compared fixation and survival of 280 lateralised vs 527 standard cementless stems after two years (1-7)	Cantin O.; Viste A.; Desmarchelier R.; Besse J.-L.; Fessy M.H. et al.	No data for collar
Clinical and Radiographic Outcomes at 25-30 Years of a Hip Stem Fully Coated With Hydroxylapatite	Jacquot L.; Bonnin M.P.; Machenaud A.; Chouteau J.; Saffarini M.; Vidalain J.-P. et al.	Fractures included
Survival and Clinical Outcomes at 30 to 35 Years Following Primary Total Hip Arthroplasty With a Cementless Femoral Stem Fully Coated With Hydroxyapatite	Jacquot L.; Machenaud A.; Bonnin M.P.; Chouteau J.; Ramos-Pascual S.; Saffarini M.; Dubreuil S.; Vidalain J.-P. et al.	Fractures included
The Corail Stem as a Reverse Hybrid – Survivorship and X-Ray Analysis at 10 Years	Wangen H, Nordsletten L, Boldt JG, Fenstad AM, Beverland DE et al.	Fractures included
Femoral stem subsidence in cementless total hip arthroplasty: a retrospective single-centre study	Ries C, Boese CK, Dietrich F, Miehlke W, Heisel C. et al.	Follow-up < 12 months
Independent predictors of failure up to 7.5 years after 35 386 single-brand cementless total hip replacements: a retrospective cohort study using National Joint Registry data	Jameson SS, Baker PN, Mason J, Rymaszewska M, Gregg PJ, Deehan DJ, Reed MR et al.	Minors
Evaluation Of Subsidence Between Collarless And Collared Corail Femoral Cement Less Total Hip Replacement	Sudhahar TA, Morapudi S, Branes K	Additional sources, not MEDLINE/Scopus indexed
