# Supplementary material for: Collared versus collarless hydroxyapatite-coated stems for primary cementless total hip arthroplasty; a systematic review of comparative studies. Is there any difference in survival, functional, and radiographic outcomes?
Source: SICOT J. 2024 Feb 15;10:8. doi: 10.1051/sicotj/2024003 (PMC10868518; doi:10.1051/sicotj/2024003)
Supplement: Supplementary file 2 — Appendix 2: Quality of included studies assessed by New-castle Ottawa scale. [file sicotj-10-8-s2.pdf]

Appendix 2

Quality of included studies assessed by New-castle Ottawa scale

Cohort studies	Selection				Comparability	Outcome			Total
Authors	Representativeness Of the exposed cohort	Selection of the non-exposed cohort	Ascertainment of exposure	Demonstration that outcome of interest was not present at start of the study	Comparability of cohorts on the basis of the design or analysis	Assessment of outcome	Was follow-up long enough for outcomes to occur	Adequacy of follow up of cohorts	
Dammerer	*	*	*	*	*	*	*	*	8
Belgaïd	*	*	*	*	*	*	*	*	8
Wirries	*	*	*	*	*	*	*	*	8
Melbye	*	*	*	*	*	*	*	-	7
Karayiannis	*	*	*	*	*	*	*	-	7

Magill	*	*	*	*	*	*	*	*	8
Hoskins	*	*	*	*	*	*	*	-	7
Louboutin	*	*	*	*	*	*	*	*	8
Magill	*	*	*	*	*	*	*	-	7
Al-Najjim	*	*	*	*	*	*	*	*	8

Risk of Bias 2 assessment

	Randomization process	Deviations from intended interventions	Missing outcome data	Measurement of the outcome	Selection of the reported result	Overall Bias
Polus	Low	Low	Low	Low	Some concerns	Low
Perelgut	Low	Low	Low	Low	Some concerns	Low
